# Upregulated GDF-15 expression facilitates pancreatic ductal adenocarcinoma progression through orphan receptor GFRAL

**DOI:** 10.18632/aging.103830

**Published:** 2020-11-17

**Authors:** Zhiping Zhao, Junfeng Zhang, Liangyu Yin, Jiali Yang, Yao Zheng, Mengjie Zhang, Bing Ni, Huaizhi Wang

**Affiliations:** 1 Institute of Hepatopancreatobiliary Surgery, Southwest Hospital, Army Medical University (Third Military Medical University), Chongqing 400038, P. R. China; 2 Institute of Hepatopancreatobiliary Surgery, Chongqing General Hospital, University of Chinese Academy of Sciences, Chongqing 400038, P. R. China; 3 Department of Pathophysiology, College of High Altitude Military Medicine, Army Medical University (Third Military Medical University), Chongqing 400038, P. R. China

**Keywords:** pancreatic ductal adenocarcinoma, GDF-15, GFRAL, proliferation, metastasis

## Abstract

Growth and differentiation factor 15 (GDF-15) has been studied as an important hallmark of cancer. However, the receptor of GDF-15 in pancreatic cancer cell remains unclear. Here, we investigated its biological effects in pancreatic ductal adenocarcinoma (PDAC). We found that aberrant GDF-15 expression positively correlated with poor survival of PDAC patients. GDF-15 protein enhanced tumor cell proliferation in two pancreatic cancer lines, AsPC-1 and BxPC-3. Knockdown GDF-15 attenuated its biological function *in vitro* and reduced PDAC cell tumorigenesis upon xenotransplantation into nude mice. Moreover, we identified that glial-derived neurotropic factor family receptor α-like (GFRAL) was upregulated in PDAC tissues and positively correlated with GDF-15 expression. High GFRAL expression was significantly associated with poor survival in PDAC patients. Furthermore, we identified that the biological effects of GDF-15 are mediated by its receptor GFRAL which is present in PDAC cells. After overexpression GFRAL in pancreatic cancer cells, the effect of GDF-15 was significantly enhanced. Overall, our findings demonstrated that the GDF-15 secreted by PDAC cells, binds to GFRAL, itself localized in PDAC cells, to promote cancer cell growth and metastasis through the GDF-15/GFRAL signaling pathway.

## INTRODUCTION

Pancreatic ductal adenocarcinoma (PDAC) has a poor prognosis largely owing to lethal malignancy, early metastasis, insignificant clinical symptoms and drug resistance [1, 2]. Overall 5-year survival rate is less than 5%, and only 10-15% of PDAC patients are eligible for surgical resection [3]. Therefore, the vast majority of patients with local progression and metastatic pancreatic cancer do not have opportunities to undergo a complete surgical resection. These patients have no other choice but to accept palliative resection [4, 5], chemotherapy [6, 7], radiotherapy [8], or immunological therapy [9]. Unfortunately, these therapeutic methods have limited clinical benefits. Therefore, it is urgent to find new early diagnostic and treatment options for PDAC.

Growth differentiation factor 15(GDF-15), also known as macrophage inhibitory cytokine-1(MIC-1), nonsteroidal anti-inflammatory drug-activated gene-1(NAG-1), prostate-derived growth factor(PDF) or placental bone morphogenetic protein(PLAB), is a distantly related member of Transforming Growth Factor-β (TGF-β) cytokine superfamily protein [10]. As one of the most divergent members of this family, GDF-15 only has 15% to 29% amino acid similarity to other members [11]. GDF-15 expression can be induced by inflammation, injury, cardiovascular diseases, obesity and malignancy [10, 12, 13]. GDF-15 is upregulated in many types of cancers, such as glioblastoma [14, 15], testicular cancer [16], ovarian cancer [17], oral squamous cell carcinoma [18], uveal melanoma [19], hepatocellular carcinoma [20], lung cancer [21], prostate, breast, colon, and gastric cancer [10, 22], including pancreatic cancer [23–28]. During the early stage of pancreatic tumorigenesis, GDF-15 can restrain tumor-associated macrophage activity by inhibiting NF-κB signaling, thereby evading macrophage immune surveillance [23]. In pancreatic cancer microenvironment, solid stress can activate Akt/CREB1 signaling pathway to upregulate GDF-15 expression, which eventually promotes tumor cell migration [26]. Twist can promote PDAC invasion and drug resistance by inducing GDF-15 expression via p38/MAPK dependent molecular mechanism [27]. Initially, data suggested that GDF-15 can interact with the receptor TGF-βRII. However, subsequent studies failed to confirm that GDF-15 acts through TGF-β receptors [29]. In mice, glial-derived neurotrophic factor receptor alpha-like (GFRAL) is highly expressed in the area postrema and can bind with GDF-15 directly to treat metabolic related diseases such as obesity, diabetes and anorexia/cachexia [29–33]. GFRAL is an orphan member of Glial-derived neurotrophic factor (GDNF) [29]. To date, four members of GDNF family proteins have been reported, GDNF family receptor-α1(GFR-α1), GFR-α2, GFR-α3 and GFR-α4, which bind to GDNF, neurturin (NRTN), artemin (ARTN), and persephin (PSPN), respectively [29, 34]. As a transmembrane protein with a very short cytoplasmic domain, GFRAL can function with its coreceptor Ret, resulting in an intracellular signaling cascade, such as through the PI3K-Akt, Erk1/2, MAPK and phospholipase C (PLC)γ pathways [29, 33, 35]. However, whether and how GFRAL is involved in the pathogenesis of pancreatic cancer is yet to be investigated.

In this study, we first discovered that GDF-15 is overexpressed in pancreatic cancer blood and tissues and that this high expression of GDF-15 is positively correlated with a poor survival of PDAC patients. Taking together, we suggest that GDF-15 could be used as a biomarker of Pancreatic Ductal Adenocarcinoma. Then, we performed several experiments, both *in vitro* and *in vivo*, to study the role and effects of GDF-15 in Pancreatic Ductal Adenocarcinoma. *In vitro*, we found that GDF-15 promotes the proliferation of several pancreatic cancer cell lines. *In vivo*, we showed that GDF-15 increases the PDAC cell tumorigenesis in nude mice. Finally, we have also shown that the biological effects of GDF-15 are mediated by its receptor GFRAL which is present in PDAC cells. Taking together, this study have demonstrated that the GDF-15 secreted by PDAC cells, binds to GFRAL, itself localized in PDAC cells, to promote cancer cell growth and metastasis.

## RESULTS

### GDF-15 is aberrantly elevated in the plasma of pancreatic cancer patients and correlates with disease progression

**Figure 1:**
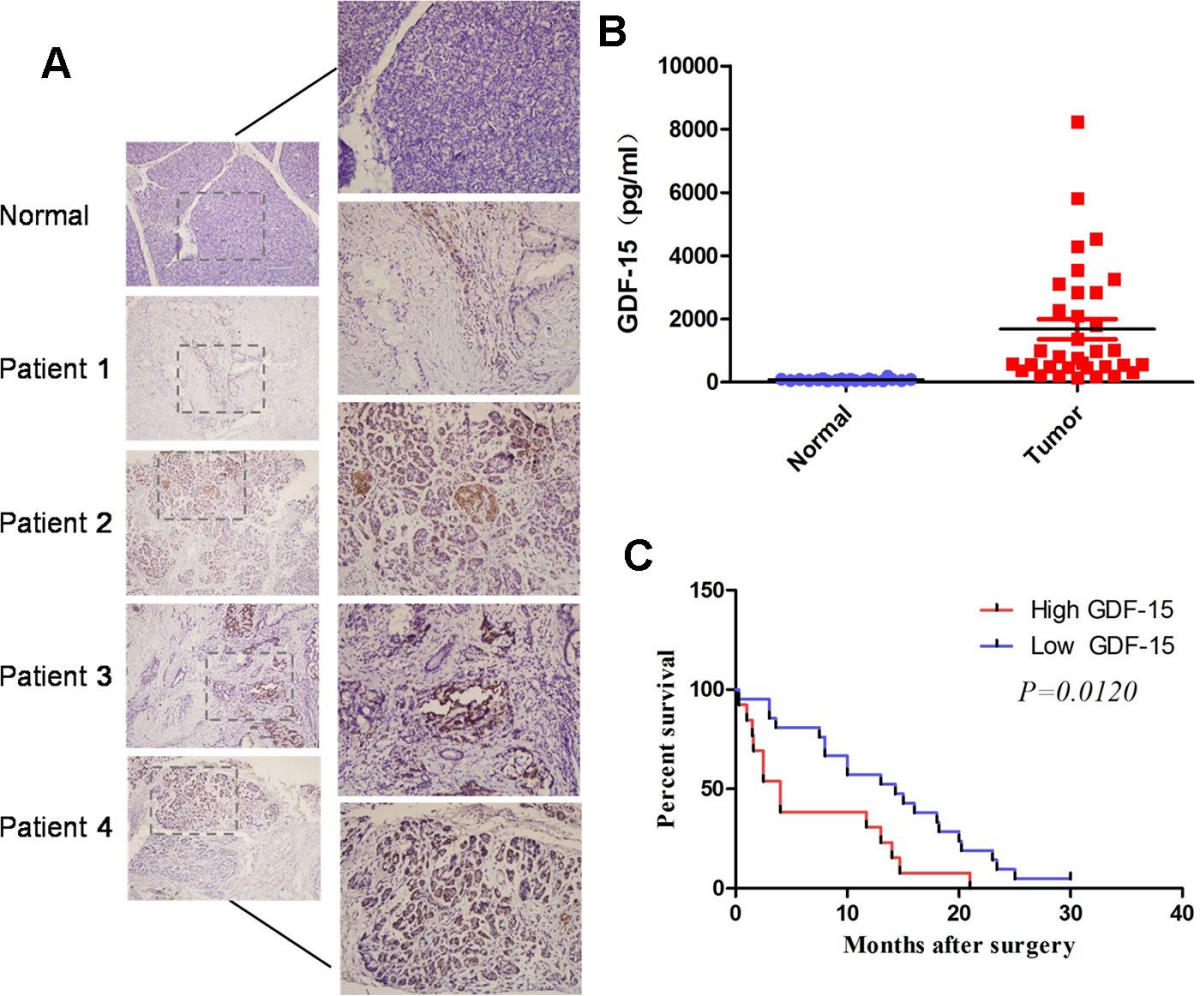
**GDF-15 is aberrantly elevated in pancreatic cancer tissues and blood, and correlates with disease progression.** (**A**) Detection of GDF-15 protein levels by immunohistochemistry (IHC) in 7 normal pancreatic tissues and 21 pancreatic cancer tissues. (**B**) GDF-15 protein expression was determined via Enzyme-Linked Immunosorbent Assay (ELISA) in 20 normal blood and 34 pancreatic cancer blood. (**C**) Kaplan-Meier curves for overall survival time of patients with pancreatic cancer after surgical resection. The high and low GDF-15 protein expression was based on the average value of GDF-15 protein expression in pancreatic cancer patients’ blood samples. ***P<0.0001.

To investigate GDF-15 expression in plasma of pancreatic cancer patients, we first performed Enzyme-Linked Immunosorbent Assays (ELISAs) to evaluate GDF-15 expression in 20 normal plasma and 34 pancreatic cancer plasma. The results indicated that GDF-15 expression was significantly elevated in pancreatic cancer blood compared with that in normal samples (Figure 1B). We also performed immunohistochemistry (IHC) assays to detect GDF-15 expression in pancreatic cancer tissues. Consistently, compared with 7 normal pancreatic tissues, GDF-15 was upregulated in 21 pancreatic cancer tissues (Figure 1A). In addition, Kaplan-Meier survival analysis showed that patients with higher GDF-15 expression had reduced 5-year overall survival rates (Figure 1C). Moreover, we found that GDF-15 expression was positively associated with tumor size and grade (Supplementary Table 2). These results suggest that the upregulation of GDF-15 might promote the progression of pancreatic cancer.

### GDF-15 promotes pancreatic cancer cell proliferation in vitro and enhances the chemosensitivity of cells to gemcitabine

**Figure 2:**
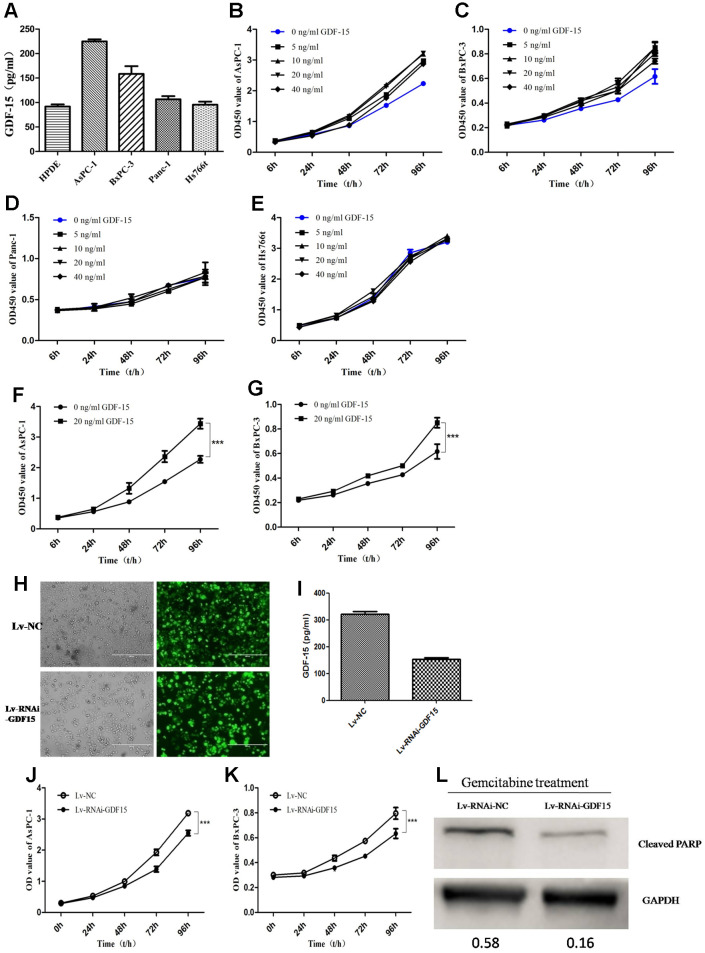
**GDF-15 promotes pancreatic cancer cell proliferation in vitro and enhances the chemosensitivity of cells to gemcitabine.** (**A**) The expression level of GDF-15 secreted by pancreatic cancer cell lines (AsPC-1, BxPC-3, Panc-1, Hs766t), was analyzed by ELISA. (**B**–**E**) Different concentrations of recombinant human GDF-15 protein, 0ng/ml, 5ng/ml, 10ng/ml, 20ng/ml, 40ng/ml, were added to the culture of the pancreatic cancer cell lines, AsPC-1, BxPC-3, Panc-1 and Hs766t. (**F**, **G**) The effect of recombinant human GDF-15 on pancreatic cancer cell lines (AsPC-1, BxPC-3) was detected by CCK-8 assay. The results are presented as the mean ± s.d. Significance was analyzed using GraphPad Prism. (**H**, **I**) The efficiency of lentivirus infection was detected by fluorescence microscope in AsPC-1 cells, and the results from ELISA assay indicated that GDF-15 expression was downregulated significantly. (**J**, **K**) The effect of GDF-15 knockdown on the proliferation of AsPC-1 and BxPC-3 cells was detected by CCK-8 assay. (**L**) WB assay for cleaved PARP in Lv-RNAi-NC- and Lv-RNAi-GDF-15-infected AsPC-1 cells after gemcitabine (20μM) for 48h using GAPDH as a loading control. ***P<0.0001.

To illuminate the biological function of GDF-15 in pancreatic cancer, we first detected GDF-15 expression in normal pancreatic epithelial cell line (HPDE) and four pancreatic cancer cell lines, AsPC-1, BxPC-3, Panc-1 and Hs766t. We found that compared with HPDE, GDF-15 was significantly higher expression in AsPC-1 and BxPC-3 cell lines, but not significantly in Panc-1 and Hs766t (Figure 2A). Subsequently, according to the reports [26, 36], different concentrations of recombinant human GDF-15 protein, 0ng/ml, 5ng/ml, 10ng/ml, 20ng/ml, and 40ng/ml, were chose to culture with the pancreatic cancer cell lines AsPC-1, BxPC-3, Panc-1 and Hs766t. Cell proliferation was significantly enhanced by GDF-15 in AsPC-1 and BxPC-3 cells (Figure 2B, 2C, 2F, 2G), but not Panc-1 and Hs766t cells (Figure 2D, 2E). These findings indicate that the cells with higher GFRAL expression are more sensitive to GDF-15(Supplementary Figure 1). Lentiviral infection system was used to knockdown GDF-15 expression (Figure 2H, 2I), the results indicate that cell proliferation was decreased significantly after knocking down GDF-15 expression in AsPC-1 and BxPC-3(Figure 2J, 2K). We assessed the migration and invasion abilities of cancer cells using Transwell and wound assays. The results showed that cell migration and invasion were significantly decreased after downregulation of GDF-15 expression (Supplementary Figure 2). In addition, to study the effects of GDF-15 on the chemosensitivity of tumor cells, gemcitabine treatment was administered after downregulating GDF-15 expression by a lentiviral infection system in AsPC-1 cells. The protein levels of the apoptosis marker Cleaved PARP was quantified by western blot analysis at 48h after gemcitabine treatment. The results showed that GDF-15 noticeably enhanced the chemosensitivity of cancer cells to gemcitabine (Figure 2L).

### GDF-15 increases tumorigenicity in vivo

**Figure 3:**
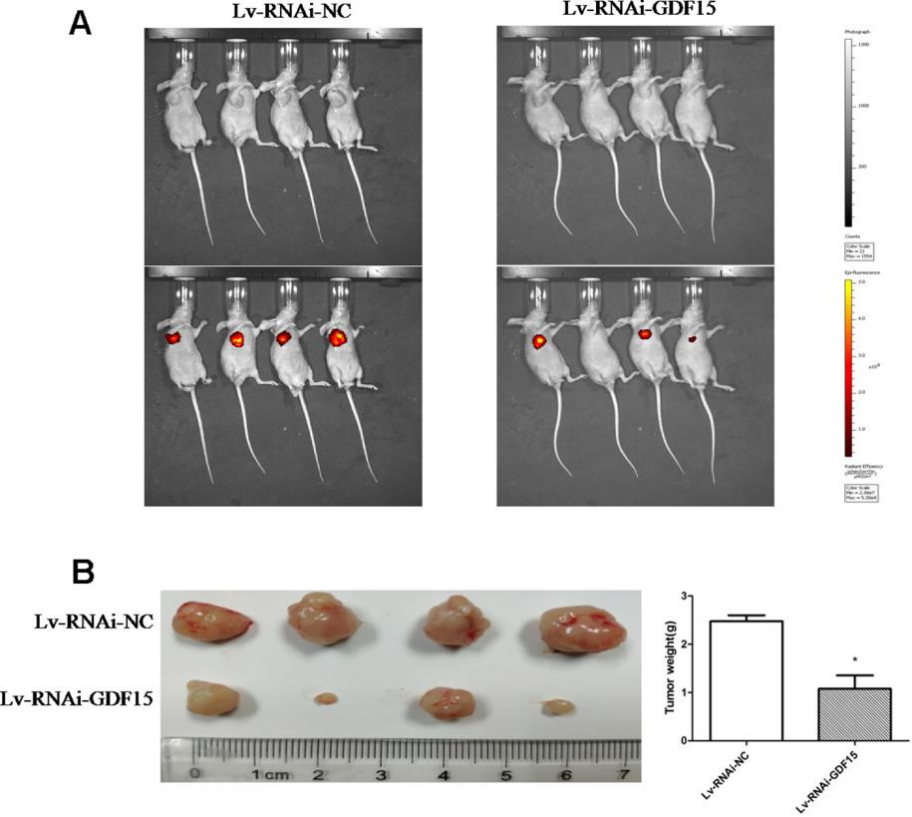
**GDF-15 promotes subcutaneous pancreatic cancer cell growth in nude mice.** Lv-RNAi-NC and Lv-RNAi-GDF-15 AsPC-1 cells were subcutaneously injected into nude mice. (**A**) Impact of GDF-15 knockdown on AsPC-1 cells growth following xenotransplantation into nude mice by subcutaneous injection. Cancer cells growth were monitored using the IVIS® Spectrum system (Perkin Elmer, USA). The intensity of red fluorescent signal means the size of tumor cells after proliferation. (**B**) The tumor growth and mean tumor weights were detected after 30 days of inoculation.

To ascertain the biological effect of GDF-15 on tumorigenicity *in vivo*, GDF-15 was downregulated via a lentiviral infection system to establish a stable pancreatic cancer cell lines, AsPC-1. We subcutaneously transplanted the AsPC-1 cells and control cells into nude mice. The tumors in the nude mice inoculated with AsPC-1 cells that were downregulated GDF-15 grew more slowly than the tumors in the control group nude mice (Figure 3A). After 30 days, the weights of tumors from AsPC-1 cells were significantly lower than the weights of tumors from control cells (Figure 3B). These findings indicate that GDF-15 can promote pancreatic cancer cell growth *in vivo*.

### GFRAL is overexpressed in pancreatic cancer and associated with a poor prognosis in pancreatic cancer patients

**Figure 4:**
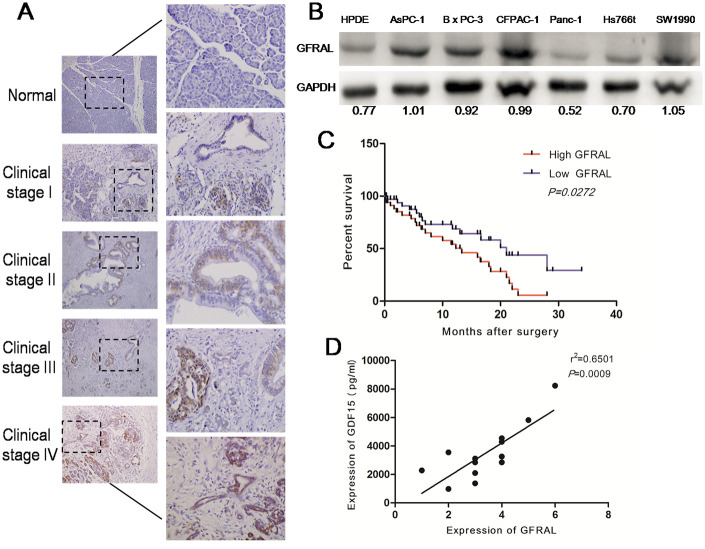
**GFRAL is overexpressed in pancreatic cancer tissues and pancreatic cancer cell lines, and correlates with disease progression.** (**A**) Detection of GFRAL protein levels by immunohistochemistry (IHC) in 117 pancreatic cancer tissues (clinical stages I, II, III, and IV) and 13 normal pancreatic tissues. (**B**) WB assay to detect GFRAL protein in different types of pancreatic cancer cell lines and normal pancreatic ductal epithelial cell line. (**C**) GFRAL expression correlates with overall survival time of patients with pancreatic cancer after surgical resection. (**D**) A positive relationship between GDF-15 and GFRAL protein was demonstrated in 13 pairs of pancreatic cancer blood and tissues based on Spearman’s correlation.

GFRAL protein expression was detected in 117 pancreatic cancer tissues and 13 normal pancreatic tissues using immunohistochemistry. GFRAL was mostly expressed on the cell membrane and the positive expression rate of GFRAL in pancreatic cancer tissues was about 94%. The results indicated that GFRAL expression was observably upregulated in pancreatic cancer tissues compared with that in normal pancreatic tissues (Figure 4A). On average, GFRAL expression was higher in stage IV pancreatic cancer tissues compared with that in stage I tissues (Figure 4A). Subsequently, we detected GFRAL protein using WB in pancreatic cell lines. The data suggested that compared with the normal pancreatic ductal epithelial cell line HPDE, GFRAL was upregulated in pancreatic cancer lines AsPC-1, BxPC-3, CFPAC-1, SW1990, but not Panc-1 or Hs766t (Figure 4B). Additionally, in order to explore whether GFRAL expression was universal in other kinds of human cancers, we detected GFRAL expression in other types of tumors, such as hepatic carcinoma, cholangiocarcinoma, colorectal carcinoma and renal clear cell carcinoma. The results showed that GFRAL protein was markedly upregulated in hepatic carcinoma, cholangiocarcinoma and colorectal carcinoma but was nearly undetectable in renal clear cell carcinoma (Supplementary Figure 3). Subsequently, Kaplan-Meier analysis revealed that patients with higher GFRAL expression had significantly lower 5-year overall survival rates (Figure 4C). Finally, we analyzed the correlation between GDF-15 and GFRAL in 13 pairs of pancreatic cancer blood and tissues. The results showed that GDF-15 expression positively correlated with GFRAL expression level (Figure 4D).

### GFRAL and GDF-15 are co-expressed in pancreatic cancer, and exhibited strong positive correlation

**Figure 5:**
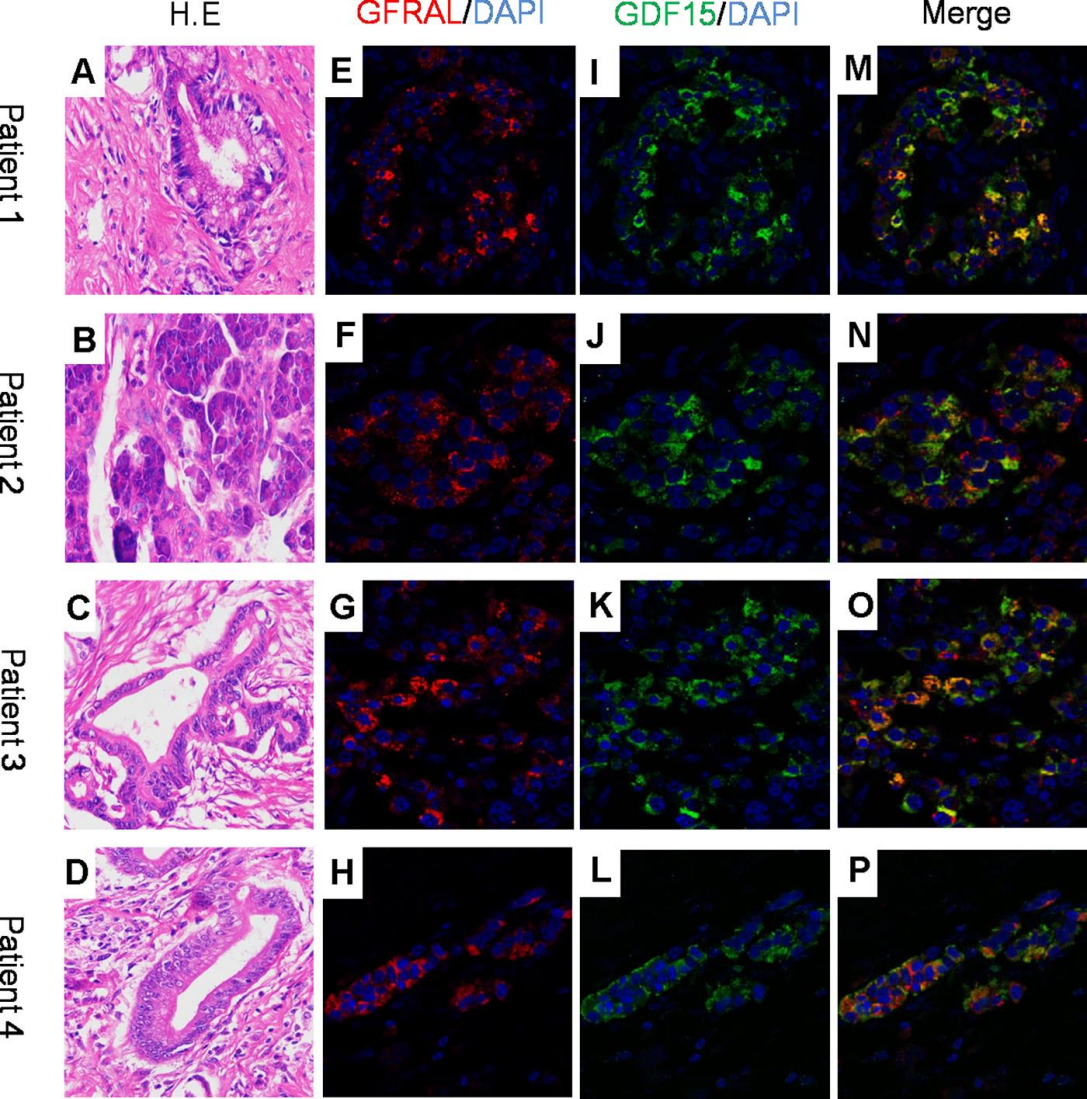
**GFRAL and GDF-15 colocalize in human pancreatic cancer cells.** (**A**–**D**) H.E images for 4 patient samples with histologically confirmed PDAC; (**E**–**H**) Immunofluorescence staining for GFRAL and nuclei counterstained with DAPI; (**I**–**L**) Immunofluorescence staining for GDF-15 and nuclei counterstained with DAPI; (**M**–**P**) Merged images of GFRAL, GDF-15, and DAPI staining.

GDF-15 has been implicated in various biological functions, such as cancer cachexia [10], angiocardiopathy and metabolism [37]. It has been reported that GFRAL protein was enriched solely in the area postrema of the brainstem of mice, rats, nonhuman primates and humans. GDF-15 can bind to GFRAL and suppress food intake, induce weight loss and improve glucose homeostasis *in vivo* in mice [29–32]. To explore whether GDF-15 plays a biological role in pancreatic cancer tissues by means of GFRAL, we performed dual immunofluorescence staining assay to detect the localization of GDF-15 and GFRAL, and found that both protein molecules are co-expressed in pancreatic ductal epithelial cancer cells (Figure 5A–5P).

### The tumor-promoting effects of GDF-15 in pancreatic cancer are mediated by the GDF-15/GFRAL pathway

**Figure 6:**
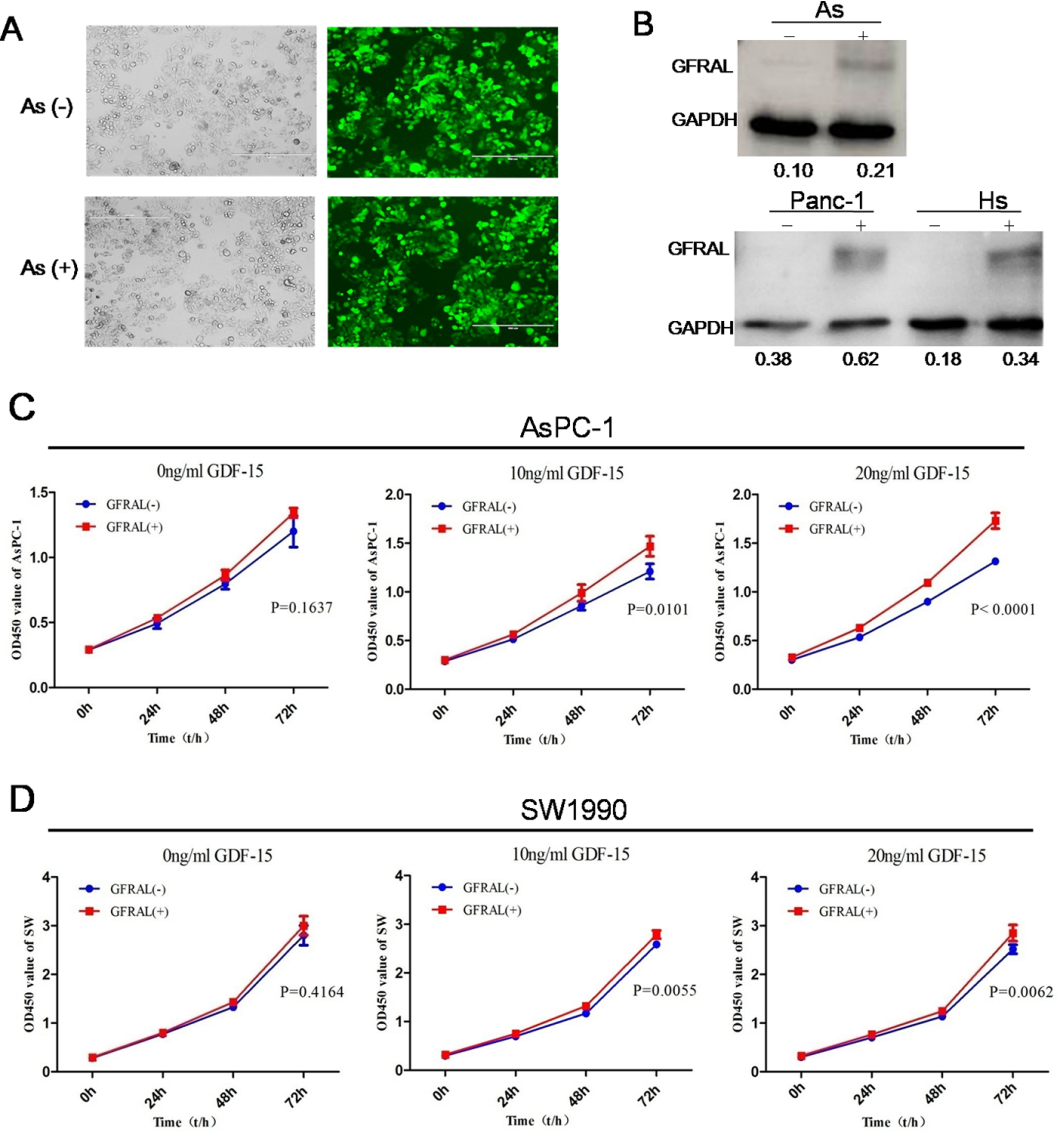
**GFRAL overexpression enhances the effects of GDF-15 in pancreatic cancer cells.** (**A**) The efficiency of lentivirus infection was detected by fluorescence microscopy in AsPC-1; As(-) means Lv-NC, As(+) means Lv-GFRAL; (**B**) WB assay indicated that GFRAL expression was significantly upregulated in different pancreatic cancer cell lines(AsPC-1, Panc-1, Hs766t). (**C**, **D**) After GFRAL upregulation in AsPC-1 and SW1990 cells, pancreatic cancer cell proliferation ability was enhanced after coculture with 0ng/ml, 10ng/ml, or 20ng/ml GDF-15, with increasing concentrations of GDF-15, the proliferative ability also increased.

Despite evidence of the tumor-promoting effects of GDF-15, the pathways through which GDF-15 mediates these effects remain unclear. It has been reported that in the area postrema of the brainstem of mice, GDF-15 can directly bind with the orphan receptor GFRAL and inhibit the behavior of food intake, leading to weight loss [29–32]. To investigate whether the GDF-15/GFRAL pathway is involved in pancreatic cancer cell progression, GFRAL protein was upregulated in AsPC-1, panc-1, Hs766t and SW-1990 cells by lentivirus transfection (Figure 6A, 6B). Cell proliferation assays showed that GFRAL overexpression significantly enhanced pancreatic cancer cell proliferation after coculture with 0ng/ml, 10ng/ml or 20ng/ml GDF-15 protein. The results showed that with increasing concentrations of GDF-15, the proliferative ability also increased (Figure 6C, 6D). Overall, these results suggest that the tumor-promoting effects of GDF-15 in pancreatic cancer are mediated by the GDF-15/GFRAL pathway.

## DISCUSSION

The development and progression of PDAC not only rely on the alterations of tumor cell itself, such as gene mutation, protein modification, epigenetic, transcriptional and metabolic changes, but also rely on the complex tumor microenvironment. In this study, we found that PDAC cells can auto secrete GDF-15 protein, which was upregulated in pancreatic cancer tissues and blood samples compared with normal human pancreas and plasma. GDF-15 expression strongly correlated with poor prognosis for pancreatic cancer patients. At the same time, compared with the normal pancreatic epithelial cell line HPDE, GDF-15 expression was upregulated in pancreatic cancer cell lines, AsPC-1 and BxPC-3. Because of the different phenotype and genotype of pancreatic cancer cell lines [38], GDF-15 expression was different between the various cancer cell lines. We also found that GDF-15 could induce pancreatic cancer cell lines AsPC-1, BxPC-3, but not Panc-1 or Hs766t to proliferate. WB assays indicated that it may be because AsPC-1 and BxPC-3 were more expressed receptor GFRAL than Panc-1 and Hs766t. Overall, our study revealed that GDF-15 secreted by PDAC cells and could promote PDAC cells growth *in vitro* and *in vivo* and enhanced chemosensitivity through directly binding to its receptor GFRAL. These findings support the important roles of GDF-15/GFRAL in promoting the development and progression of pancreatic cancer.

We perused the PubMed/Nucleotide database and found that GDF-15 was located on the human chromosome 19, GRCh38.p13 and composed of 7007bp linear DNA. Its NCBI Reference Sequence was NC_000019.10. It was reported that GDF-15 was expressed in various diseases and played different biological roles. In diabetic kidney disease (type 2 diabetes with nephropathy), serum GDF-15 was a promising biomarker of renal functional decline [39]. GDF-15 serum levels were elevated in Egyptian systemic sclerosis patients, and it could be a treatment target [40]. In chronic obstructive pulmonary disease, GDF-15 contributes independently to subclinical coronary atherosclerosis [41]. After cigarette exposure, GDF-15 was elevated and could contribute to pulmonary inflammation [42]. GDF-15 could act as a novel biomarker in cardiovascular disease [43, 44]. Serum GDF-15 was significantly higher in rheumatoid arthritis patients and correlated with disease activity and subclinical atherosclerosis [45]. GDF-15 was expressed in visceral and subcutaneous adipose tissue in obese patients and played a role in the modulation of adipose tissue function and body fat mass [46]. More recently, Pavo et al. reported that GDF-15 is associated with cancer incidence in patients with type 2 diabetes [37].

GDF-15 overexpression has been observed in several types of tumor disease, such as glioblastoma [14, 15], testicular cancer [16], ovarian cancer [17], oral squamous cell carcinoma [18], uveal melanoma [19], hepatocellular carcinoma [20], lung cancer [21], prostate, breast, colon, and gastric cancer [10, 22], including pancreatic cancer [23–28]. GDF-15 concentration in serum could be considered a biomarker in differentiating pancreatic mass due to chronic pancreatitis from pancreatic adenocarcinoma [25]. GDF-15 was also associated with cancer-associated weight loss [24].

Secreted GDF-15 could inactivate tumor infiltrating macrophages, thus evading macrophage immune surveillance and allowing the expansion of pancreatic cancer [23].

GFRAL was recently identified as a type of central nervous system receptor for mediating the anorectic actions of GDF-15, which could cause anorexia and weight loss in mice [47]. In issues of *Nature Medicine* and *Nature*, four different medical research groups, Emmerson PJ [29], Yang L [30], Mullican SE [31], and Hsu JY [32], have reported that GFRAL was highly expressed in the area postrema and could bind with GDF-15 directly to treat metabolic related diseases such as obesity, type 2 diabetes and anorexia, in mice. So far, there is no related study describing the function of GFRAL in pancreatic cancer. Therefore, we performed immunohistochemistry and detected that GFRAL was upregulated in human pancreatic cancer tissues. Additionally, in order to explore whether GFRAL expression was universal in other kinds of human cancers, we performed IHC and found that GFRAL protein was markedly upregulated in hepatic carcinoma, cholangiocarcinoma and colorectal carcinoma but was nearly undetectable in renal clear cell carcinoma. It may be because of tissue specificity of gene expression. Furthermore, Kaplan-Meier analysis indicated that GFRAL was closely related to a significant reduction in overall survival rates. On the other hand, we also found GDF-15 was upregulated in pancreatic cancer plasma and tissues. Pancreatic cancer cell proliferation and migration were significantly changed by the knockdown and upregulation of the expression of the two proteins GDF-15 and GFRAL. Our findings, together with previous evidence, indicate that GDF-15/GFRAL axis can promote pancreatic cancer proliferation and migration. However, the signal pathway in pancreatic cancer needs further exploration.

In conclusion, we demonstrated that PDAC cells can auto secrete GDF-15 protein, which can directly bind with its membrane receptor GFRAL to promote cancer cell progression. The newly identified GDF-15/GFRAL axis provides a vital and novel potential biomarker for diagnosis and therapeutics in pancreatic cancer.

## MATERIALS AND METHODS

### Tissue and blood samples

Pancreatic cancer and blood samples were obtained from the Institute of Hepatopancreatobiliary Surgery, Southwest Hospital, Third Military Medical University (Army Medical University). The normal pancreatic and blood samples were obtained from organ donors. These pancreatic samples had been fresh-frozen in liquid nitrogen at the time of postoperative. All of these samples were categorized by Patient ID. None of the patients had received radiotherapy or chemotherapy before surgery. The experimental human tissues were approved by the Ethics Committee of Southwest Hospital.

### Cell culture and transfection

The normal human pancreatic ductal epithelial cell line HPDE and the human pancreatic cancer cell lines AsPC-1, BxPC-3, CFPAC-1, Panc-1, SW1990 and Hs766t (ATCC, USA) were cultured incomplete growth medium of DMEM supplemented with 10% fetal bovine serum (FBS; Invitrogen) and 1% penicillin/streptomycin (Sigma, St. Louis, MO, USA), which was recommended by the manufacturer. Cultured cells were maintained in a humidified 5% CO_2_ atmosphere at 37 °C. We got lentivirus vectors LV-GDF15-RNAi and negative viral vector CON053 of GDF-15 gene RNA interference from Shanghai Genechem Company. The LV-GDF15-RNAi target sequence was CCGGATACTCACGCCAGAAGT. Flow cytometry was used to sort cells transfected with lentivirus LV-GDF15-RNAi.

### Cell proliferation assay

Pancreatic cancer cells were cultured in 96-well plates at a density 2×10^3^/well in 100μl/well. Cell proliferation was measured by a Cell Counting Kit-8 (CCK-8) (Dojindo, Kumanoto, Japan) according to the manufacturer’s instructions. The OD450 was measured by spectrophotometry(Bio Tek, Vermont, USA) after a 2-hour incubation with 20μl of CCK-8 reagent.

### Transwell assay

Pancreatic cancer cells were suspended in serum-free DMEM and adjusted the cell number to 2×10^5^/well in 400μl/well in the top chamber (24-well insert, 8-μm pore size, Millipore, Massachusetts, USA), which was coated with a layer of extracellular matrix (BD Biosciences, USA). DMEM supplemented with 10% fetal bovine serum (FBS; Invitrogen) and 1% penicillin/streptomycin (Sigma, St. Louis, MO, USA) (total 500 ml) were added to the bottom chamber. Pancreatic cancer cells which had invaded through the extracellular matrix to the lower surface of the filter were stained after 20-hour incubation in a humidified 5% CO_2_ atmosphere at 37 °C. Three independent replicates of each experiment were performed.

### Wound assay

The wound assays were performed using previously described methods [48]. Pancreatic cancer cells were seeded in a 6-well plate, grown until confluence, and then starved for 24 h. Monolayer of cells were scratched using a sterile plastic tip. Images were measured at different time points by photographing the wound in three random fields.

### ELISA

GDF-15 levels in human blood samples and supernatants of pancreatic cancer cells were measured using a standard ELISA (R&D Systems, catalog #DGD150), following the manufacturer’s instructions. The mean value was used to differentiate high and low GDF-15 serum levels group.

### Immunohistochemistry (IHC) and Immunofluorescence (IF)

Consecutive 5-μm sections were cut from paraffin-embedded samples. For antigen retrieval, the deparaffinized sections were boiled for 2 minutes in citrate buffer, pH 6.0. Endogenous peroxidase activity was blocked through incubation with 3% hydrogen peroxide solution for 30 minutes at room temperature. The primary antibody was hybridized with the tissue samples at 4°C overnight followed by incubation with a secondary antibody. The staining results were evaluated by two experienced pathologists in a double-blinded manner and quantified using a composite score obtained by multiplying the values of staining intensities (0, no staining; 1, weak staining; 2, moderate staining; 3, strong staining) and the percentage of positive cells (0, 0%; 1, <10%; 2, 10–50%; 3, >50%). The antibodies used for those studies are described in the Supplementary Table 1.

### Protein extraction and Western blotting

Pancreatic cancer cell lines (AsPC-1, BxPC-3, CFPAC-1, Panc-1, SW-1990 and Hs766t) were seeded in 6-well plates. After 48 h, protein extracts were separated by electrophoresis in a sodium dodecylsulfate-polyacrylamide gel (Invitrogen, Camarillo, CA, USA) and transferredonto polyvinylidene fluoride membranes (Millipore, Billerica, MA, USA) for immunoblotting. The membranes were hybridized with a primary antibody at 4 °C overnight followed by incubation with a secondary antibody for 1h at room temperature. Glyceraldehyde-3-phosphate dehydrogenase was used as the loading control. Signals were visualized using a chemiluminescent detection system (Thermo Scientific, Rockford, IL, USA) and exposure to film. The antibodies used for those studies are described in the Supplementary Table 1.

### Xenograft study

Female athymic nude mice, 4 to 6 weeks old, were purchased from the Peking University Animal Center (Beijing, China). All of the immunodeficient mice were from the same batch and were homogeneous. After 5 days of acclimatization, a total of 2 × 10^6^ AsPC-1 cells stably transfected with the lentivirus which was performed using a shRNA sequence targeting GDF-15, or the negative control was injected subcutaneously into the right axilla of each mouse. Mice are anesthetized by isoflurane gas on the 30^th^ day after injection and the proliferation of subcutaneous tumors in nude mice was detected by the IVIS® Spectrum in vivo imaging system (Perkin Elmer, USA). The intensity of red fluorescent signal means the size of tumor cells after proliferation. The mice were sacrificed on the 30^th^ day after injection. Tumor volume was calculated using the equation (L ×W^2^)/2, and the tumor weights were measured and recorded in grams. The volume and weights of tumor stand for PDAC AsPC-1 cells’ growing situation *in vivo*. All animal studies were approved by the Institutional Animal Care and Use Committee of the Third Military Medical University (Army Medical University).

### Statistical analysis

All experiments were repeated at least three times and all data were analyzed using SPSS 17.0 statistical software (version17.0, Chicago, IL, USA). All grouped data were presented as the means± SDs. One-way or two-way ANOVA was used for comparisons between groups. P values below 0.05 were considered statistically significant.

## Supplementary Material

Supplementary Figures

Supplementary Tables
