# Supplementary material for: Upregulated GDF-15 expression facilitates pancreatic ductal adenocarcinoma progression through orphan receptor GFRAL
Source: Aging (Albany NY). 2020 Nov 17;12(22):22564–81. doi: 10.18632/aging.103830 (PMC7746332; doi:10.18632/aging.103830)
Supplement: Supplementary Figures [file aging-12-103830-s001..pdf]

SUPPLEMENTARY FIGURES

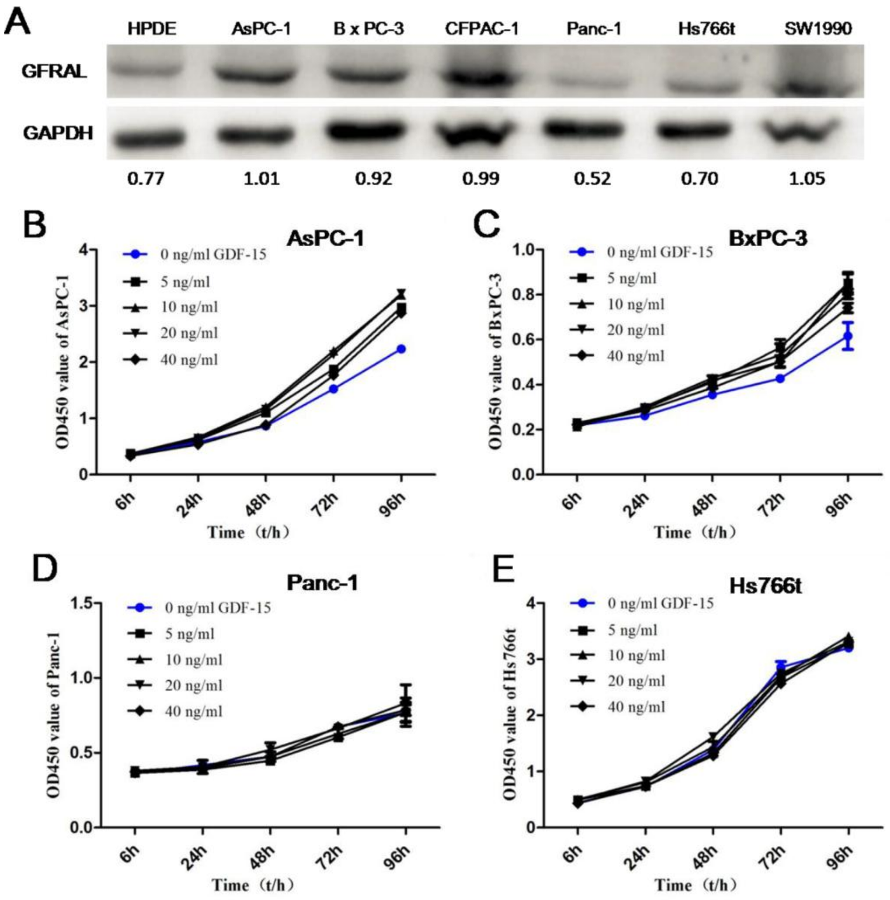

Supplementary Figure 1. Pancreatic cancer cells with higher GFRAL expression are more sensitive to GDF-15 protein. (**A**) WB assays find that, compared with the normal pancreatic cell HPDE, GFRAL protein is upregulated in AsPC-1, BxPC-3, and downregulated in Panc-1, Hs766t. (**B**, **C**) Pancreatic cancer cells ,AsPC-1 and BxPC-3, are sensitive to GDF-15 protein. (**D**, **E**) Panc-1 and Hs766t cells are insensitive to GDF-15 protein.

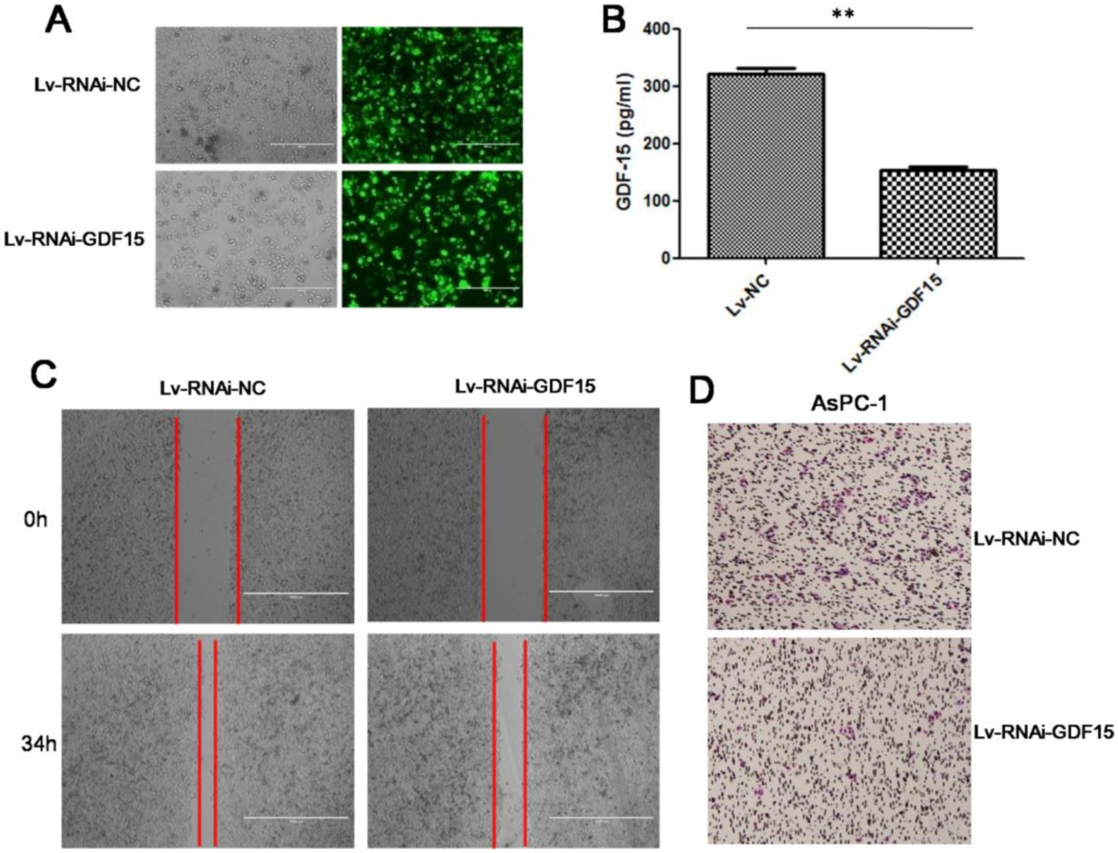

Supplementary Figure 2. Pancreatic cancer cell AsPC-1 migration and invasion were significantly decreased after downregulation of GDF-15 expression. (**A**) The efficiency of lentivirus infection was detected by fluorescence microscope in AsPC-1 cells. (**B**) ELISA assays indicated that GDF-15 expression was downregulated by LV-RNAi-GDF15 significantly. (**C**, **D**) Wound and transwell assays showed that cell migration and invasion were significantly decreased after downregulation of GDF-15 expression.

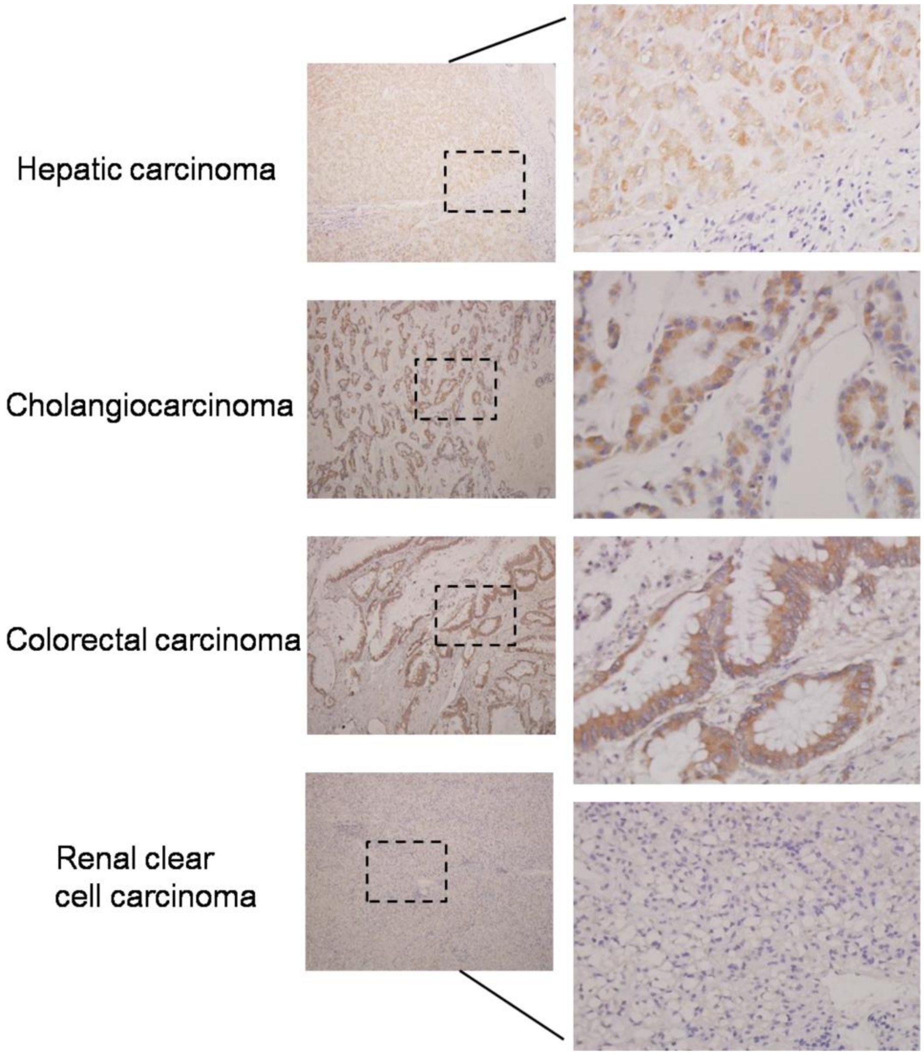

Supplementary Figure 3. Immunohistochemistry (IHC) assays showed that GFRAL protein is expressed in hepatic carcinoma, cholangiocarcinoma, colorectal carcinoma tissues, but renal clear cell carcinoma tissues.
