# Supplementary material for: Upregulated GDF-15 expression facilitates pancreatic ductal adenocarcinoma progression through orphan receptor GFRAL
Source: Aging (Albany NY). 2020 Nov 17;12(22):22564–81. doi: 10.18632/aging.103830 (PMC7746332; doi:10.18632/aging.103830)
Supplement: Supplementary Tables [file aging-12-103830-s002..pdf]

SUPPLEMENTARY TABLES

Supplementary Table 1. Antibodies used for immunohistochemistry, immunofluorescence and western blot.

Antibody	Company/Provider
anti-GDF-15	Abcam plc, Cambridge, UK
anti-GFRAL	Abcam plc, Cambridge, UK
anti-Cleaved PARP	Abcam plc, Cambridge, UK
anti-GAPDH	Santa Cruz Biotechnology, CA, USA
Goat Anti-Mouse IgG H&L (Alexa Fluor® 488)	Abcam plc, Cambridge, UK
Goat Anti-Rabbit IgG H&L (Alexa Fluor® 555)	Abcam plc, Cambridge, UK
Goat Anti-Rabbit IgG-HRP	Santa Cruz Biotechnology, CA,USA
Goat Anti-Mouse IgG-HRP	Santa Cruz Biotechnology, CA,USA

Supplementary Table 2. Clinical features of pancreatic ductal adenocarcinoma (PDAC) patients (N = 34) according to GDF-15 expression.

	GDF-15 expression		P
	Low group	High group	
	20	14	
Age			0.53
>58 (n=21)	12	9	
≤58 (n= 13)	8	5	
Gender			0.71
Female (n=15)	7	8	
Male (n=19)	13	6	
Grade			0.11
G1 (n= 9)	6	3	
G2 (n=18)	10	8	
G3 (n= 7)	4	3	
Tumor Size			0.45
>3cm (n=16)	8	8	
≤3cm (n= 18)	12	6	
Overall Survival			<0.01
>1 years (n= 15)	11	4	
≤ 1 years (n=19)	9	10	
